# Identification of two novel mutations in the *SLC45A2* gene in a Hungarian pedigree affected by unusual OCA type 4

**DOI:** 10.1186/s12881-017-0386-7

**Published:** 2017-03-15

**Authors:** Lola Tóth, Beáta Fábos, Katalin Farkas, Adrienn Sulák, Kornélia Tripolszki, Márta Széll, Nikoletta Nagy

**Affiliations:** 1 Department of Medical Genetics, University of Szeged, 6 Somogyi Bela Street, 6720 Szeged, Hungary; 2 Mór Kaposi Teaching Hospital of the Somogy County, Kaposvár, Hungary; 3 MTA-SZTE Dermatological Research Group, University of Szeged, Szeged, Hungary; 4 Department of Dermatology and Allergology, University of Szeged, Szeged, Hungary

**Keywords:** Oculocutaneous albinism type 4, Unusual phenotype, *SLC45A2* gene, Compound heterozygous state, Novel mutations

## Abstract

**Background:**

Oculocutaneous albinism (OCA) is a clinically and genetically heterogenic group of pigmentation abnormalities. OCA type IV (OCA4, OMIM 606574) develops due to homozygous or compound heterozygous mutations in the *solute carrier family 45, member 2* (*SLC45A2*) gene. This gene encodes a membrane-associated transport protein, which regulates tyrosinase activity and, thus, melanin content by changing melanosomal pH and disrupting the incorporation of copper into tyrosinase.

**Methods:**

Here we report two Hungarian siblings affected by an unusual OCA4 phenotype. After genomic DNA was isolated from peripheral blood of the patients, the coding regions of the *SLC45A2* gene were sequenced. In silico tools were applied to identify the functional impact of the newly detected mutations.

**Results:**

Direct sequencing of the *SLC45A2* gene revealed two novel, heterozygous mutations, one missense (c.1226G > A, p.Gly409Asp) and one nonsense (c.1459C > T, p.Gln437*), which were present in both patients, suggesting the mutations were compound heterozygous. In silico tools suggest that these variations are disease causing mutations.

**Conclusions:**

The newly identified mutations may affect the transmembrane domains of the protein, and could impair transport function, resulting in decreases in both melanosomal pH and tyrosinase activity. Our study provides expands on the mutation spectrum of the *SLC45A2* gene and the genetic background of OCA4.

## Background

Oculocutaneous albinism (OCA) is a clinically and genetically heterogenic group of rare monogenic diseases characterized by reduced melanin production in the skin, hair and/or eyes [1]. OCA symptoms can include poor visual acuity, nystagmus, iris transillumination, strabismus, photophobia, foveal hypoplasia and misrouting of optic nerve fibers at the chiasm [2]. All OCA forms exhibit autosomal recessive inheritance [1].

OCA type 4 (OCA4, OMIM 606574) is a rare form of OCA caused by mutations in the *solute carrier family 45, member 2* (*SLC45A2*) gene on chromosome 5p13 [3]. The *SLC45A2* gene encodes a membrane-associated transport protein (MATP), which is located in melanosomes and shows high sequence and structural similarity to *Drosophila melanogaster* and plant sucrose transporters containing an RXGRR motif [4, 5]. *SLC45A2* knockdown reduced melanin content and tyrosinase activity by acidifying the pH of melanosomes in a human melanoma cell line, MNT-1 [6]. It has been suggested that, as a proton/sugar symporter, MATP transports sugars from the melanosomes to the cytoplasm using a proton gradient generated by a proton pump. Thus, normal protein function ensures elevated melanosomal pH, allowing proper binding of copper to tyrosinase and resulting in normal tyrosinase activity [6].

To date, 78 of the mutations identified in the *SLC45A2* gene are related to OCA4 [7]. In this study, we report a Hungarian family with two members affected by OCA4. Our genetic investigation identified that these members carried two novel heterozygous mutations in a compound heterozygous state, expanding the mutational spectrum of OCA4.

## Methods

### Patients

**Fig. 1:**
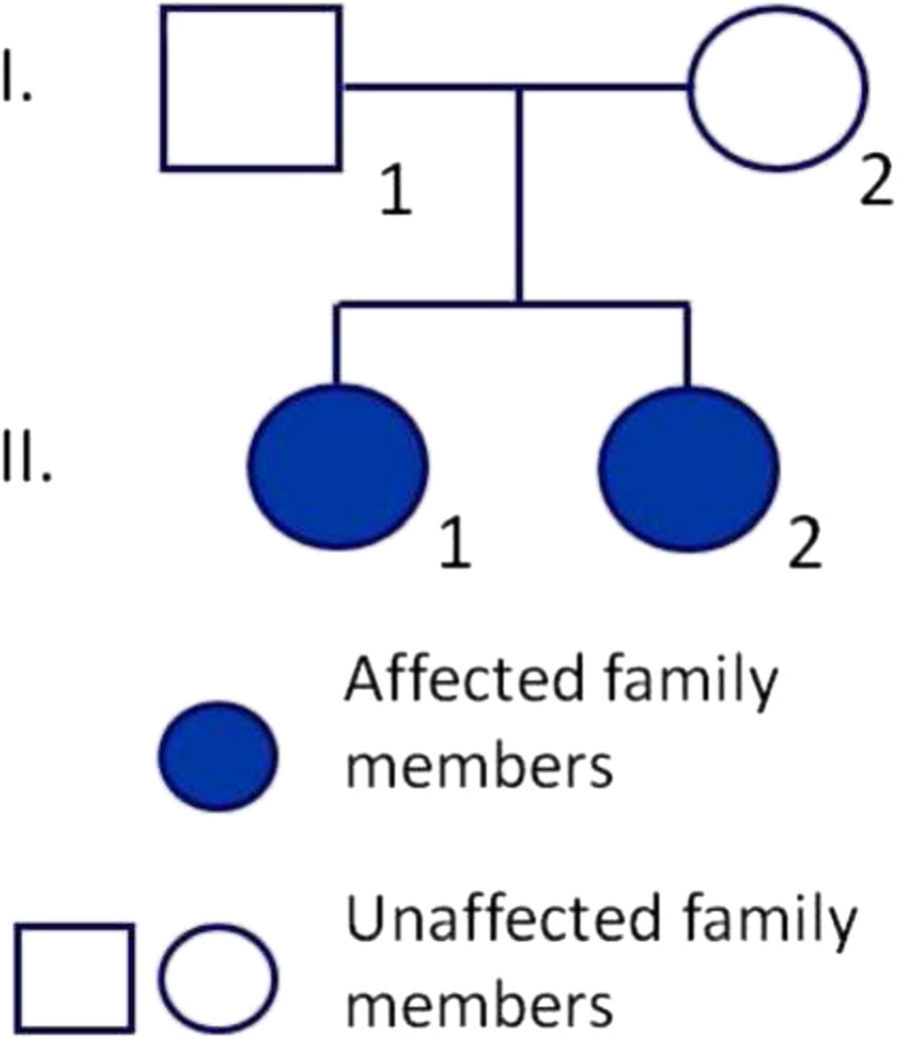
Pedigree of the patients. The investigated Hungarian pedigree spans two generations and includes two affected siblings (II/1 and II/2)

A Hungarian family with two affected siblings was investigated (Fig. 1). The affected individuals were 30 (Patient II/1) and 27 years (Patient II/2) old at the time of investigation. Both exhibited pale skin, complete absence of hair pigment, pink nevi and blue eyes with nystagmus. This complete absence of pigmentation is unusual for OCA4. Patient II/1 has been suffering from Crohn’s disease for 9 years and hypothyreosis for 4 years. Patient II/2 was not aware of any known concomitant diseases. The parents (I/1 and I/2) of the affected siblings are clinically unaffected by OCA4. The investigated patients declined publication of their clinical pictures.

### Genetic investigation

Blood was taken from the affected patients as well as from unrelated, healthy Hungarian individuals without pigmentation abnormality (*n* = 30), and genomic DNA was isolated using a BioRobot EZ1 DSP Workstation (QIAGEN; Godollo, Hungary). The entire coding region of the *SLC45A2* gene and the flanking introns were amplified and sequenced (primer sequences used were taken from the UCSC Genome Browser www.genome.ucsc.edu). The investigation was approved by the Internal Review Board of the University of Szeged. Written informed consent was obtained from the patient and the study was conducted according to the Principles of the Declaration of Helsinki. After identifying the causative mutations in the patients, further genetic screening of the parents was declined.

### Pathogenicity predictions for missense variants

In silico tools were applied to identify the functional role of the newly found variants. Here we used SIFT (Sorting Intolerant from Tolerant, http://sift.jcvi.org/), PolyPhen2 (Polymorphism Phenotyping, http://genetics.bwh.harvard.edu/pph2), Mutation Taster (http://www.mutationtaster.org/), PredictSNP (http://loschmidt.chemi.muni.cz/predictsnp/) PROVEAN (Protein Variation Effect Analyzer, http://provean.jcvi.org/index.php) and PANTHER (Protein ANalysis THrough Evolutionary Relationships, http://www.pantherdb.org/) tools.

## Results

**Fig. 2:**
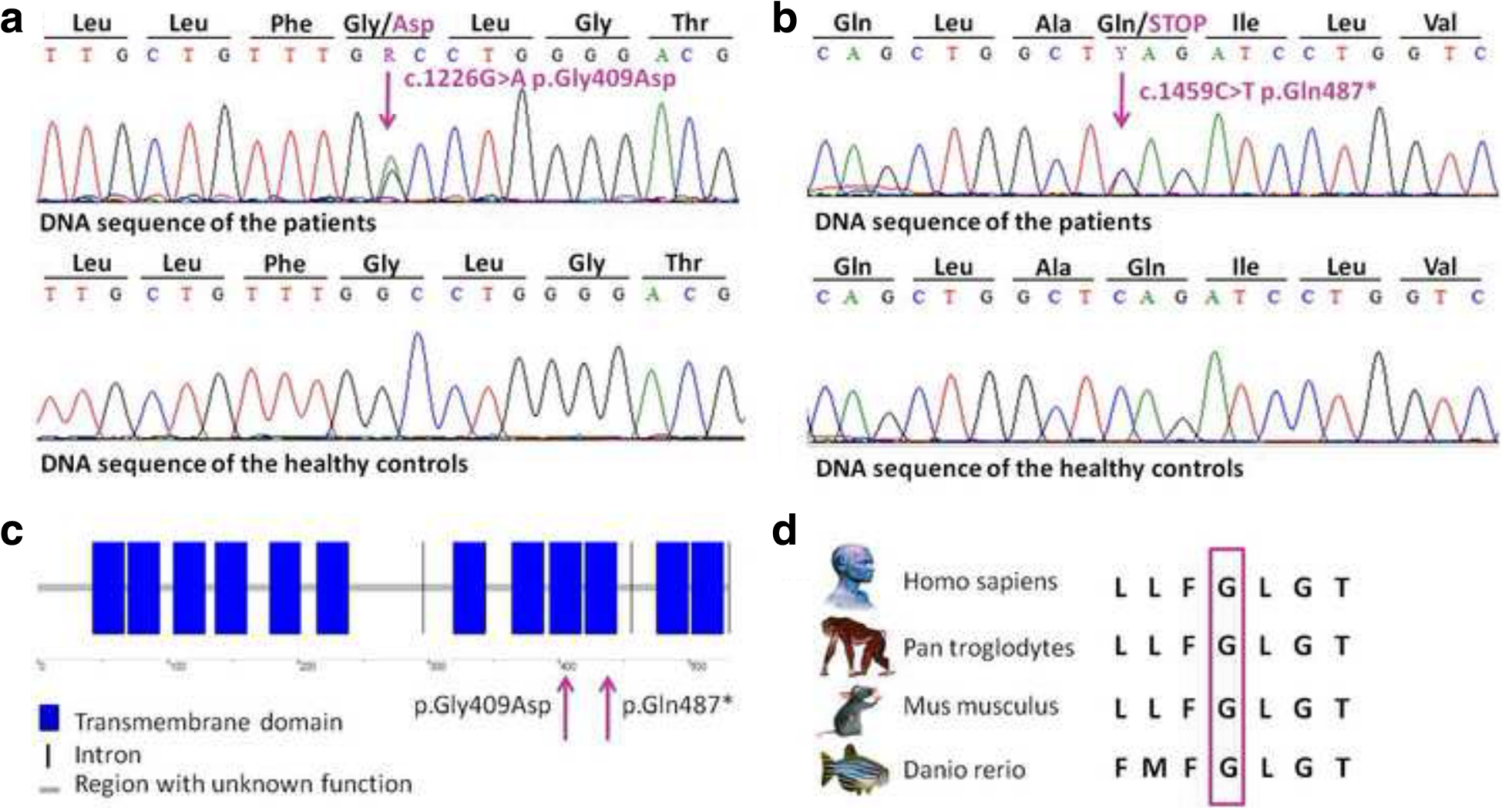
Identification of two novel mutations of the *SLC45A2* gene. **a** Direct sequencing revealed a heterozygous missense mutation (c.1226G > A, p.Gly409Asp) in the sixth exon and **b** a heterozygous nonsense mutation (c.1459C > T, p.Gln437*) in the seventh exon of the gene. Both mutations were present in both affected patients. Unrelated controls (*n* = 30) carried the wild type sequence. **c** The identified mutations are located within the transmembrane domains of the MATP protein. **d** The identified missense mutation is situated within an evolutionary conserved region

Direct sequencing of the coding regions and the flanking introns of the *SLC45A2* gene revealed two heterozygous mutations, one missense mutation (c.1226G > A, p.Gly409Asp) in the sixth exon (Fig. 2a) and one nonsense mutation (c.1459C > T, p.Gln437*) in the seventh exon (Fig. 2b). Both patients carried both mutations, suggesting a compound heterozygous state. Unrelated healthy controls carried the wild type sequence. To decide, whether the detected missense mutation is pathogenic, we use in silico analysis tools (SIFT, PolyPhen2, Mutation Taster, PredictSNP, PROVEAN and PANTHER). All prediction tools suggested that the p.Gly409Asp mutation is deleterious. The nonsense mutation was deemed to be pathogenic. It causes the development of a premature termination codon at 487 amino acid position thereby the MATP protein truncated and it may lead to its dysfunction.

## Discussion

Both mutations are situated in transmembrane domains of the MATP protein (Uniprot: Q9UMX9): the p.Gly409Asp missense mutation is located within the ninth domain and the p.Gln437* nonsense mutation within the tenth (Fig. 2c). The locations of the mutations suggest that they impair the transport function of the MATP protein. MATP dysfunction might cause an acidic melanosomal lumen, leading to improper incorporation of copper into typrosinase. The reduced tyrosinase activity could, in turn, lead to the development of the OCA phenotype [6]. The p.Gly409Asp missense mutation affects an evolutionary conserved region of the MATP protein (Fig. 2d), further emphasizing the putative pathogenic role of this mutation in the development of the observed pigmentation abnormalities of the affected patients.

Mutations of the *SLC45A2* gene have been reported to cause complete or partial loss of pigmentation, thus contributing to the development of several different OCA phenotypes. However, a genotype–phenotype correlation based the *SLC45A2* mutations and the patients’ clinical symptoms has not yet been established for OCA4 [8]. Mutations of the *SLC45A2* gene are typically associated with partial loss of pigmentation, referred to as the “brown OCA” phenotype [7]. The two siblings reported here exhibited an unusual OCA4 phenotype, as they developed the complete absence of pigmentation. This phenotype is more common in type 1 OCA, which is caused by mutations in the *tyrosinase (TYR)* gene. To rule out the influence of other putative genetic-modifier variants responsible for the unusual phenotype, the mutation screening of the *TYR* and *OCA2* genes was also performed. Common polymorphisms of *TYR* gene were detected, neither pathogenic nor non-pathogenic variants of the *OCA2* gene were identified. Patient II/1 carried the p.Ser192Tyr variant homozigously and the p.Arg402Gln variant heterozigously. Heterozygous p.Ser192Tyr polymorphism was identified in Patient II/2. These two common variants of *TYR* gene occur at high frequency (p.Ser192Tyr: Global MAF: 0.1234, Caucasian MAF: 0.3718; p.Arg402Gln: Global MAF: 0.0813, Caucasian MAF: 0.2525) but were not directly related to pigmentation phenotypes in normal Caucasians [9]. However, functional studies reported that 192Tyr and 402Gln alleles have reduced TYR enzyme activity. Heterozygous p.Ser192Tyr and p.Arg402Gln variants caused significant reduction in TYR expression, and a consistent decrease in TYR protein levels was observed in homozygous p.Ser192Tyr cells [9].

Since one of our investigated OCA4 patients (II/1) is also affected by Chron’s disease, it is possible that the mutations of the *SLC45A2* gene could be susceptibility factors for the development of Chron’s disease. This hypothesis is further supported by the literature, since a previous study reporting a sister and a brother affected by congenital neutropenia and oculocutaneous albinism identified a nonsense mutation in the *G6PC3* gene (c.829C > T, p.Gln277*) responsible for the development of congenital neutropenia and frameshift mutation in the *SLC45A2* gene (c.986delC, p.T329Rfs*68), which could explain the OCA phenotype [10]. In this previous study, the investigated brother is also affected by Chron’s disease, suggesting a putative association between the mutations of the *SLC45A2* gene and Chron’s disease [10].

OCA has been considered for many years as a group of monogenic rare diseases without cure. Accumulating knowledge regarding the underlying mechanism of the OCA4 might alter this viewpoint: it has been recently demonstrated in MNT-1 cell lysates that exogenously applied copper recovers reduced tyrosinase activity resulting from *SLC45A2* knockdown [6].

## Conclusions

In conclusion, we report two novel heterozygous mutations, one missense and one nonsense, of the *SLC45A2* gene in two Hungarian sisters affected by OCA4. The prediction analysis and the location of the mutations as well as the evolutionary conservation of the missense mutation suggest a pathogenic role in the development of OCA4. Our report, which further contributes to the mutation spectrum of the *SLC45A2* gene as well as to the spectrum of the observed unusual clinical symptoms, will hopefully contribute to future studies characterizing genotype-phenotype correlations in OCA4. This study provides expands to the genetic background of OCA4 and might serve as a basis for future studies aiming to develop novel therapeutic approaches for OCA patients.

## Abbreviations

*G6PC3*: Glucose 6 phosphatase, catalytic, 3 gene
MATP: Membrane-associated transport protein
MNT-1: Human melanoma cell line
OCA: Oculocutaneous albinism
*OCA2*: Oculocutaneous albinism II gene
OCA4: Oculocutaneous albinism type IV
RXGRR: A conserved amino acid motif
SCN4: Severe congenital neutropenia type 4
*SCN4*: Sodium channel, voltage gated, type IVgene
*SLC45A2*: Solute carrier family 45, member 2 gene
*TYR*: Tyrosinase gene
